# From Variability to Standardization: The Impact of Breast Density on Background Parenchymal Enhancement in Contrast-Enhanced Mammography and the Need for a Structured Reporting System

**DOI:** 10.3390/cancers17152523

**Published:** 2025-07-30

**Authors:** Graziella Di Grezia, Antonio Nazzaro, Luigi Schiavone, Cisternino Elisa, Alessandro Galiano, Gatta Gianluca, Cuccurullo Vincenzo, Mariano Scaglione

**Affiliations:** 1 Department of Radiology, Link Campus University, 00165 Rome, Italy; 2 Independent Researcher, 83100 Avellino, Italy; info@antonionazzaro.it; 3 Department of Radiology, University of Campania “Luigi Vanvitelli”, 80138 Naples, Italy; schiavoneluigi.96@gmail.com (L.S.); gianluca.gatta@unicampania.it (G.G.); 4 Department of Radiology, P.O. ‘A. Perrino’ Hospital, 72100 Brindisi, Italy; elisaciste@gmail.com (C.E.); alessgaliano@yahoo.it (A.G.); 5 Department of Nuclear Medicine, University of Campania “Luigi Vanvitelli,” 80138 Naples, Italy; vincenzo.cuccurullo@unicampania.it; 6 Department of Radiology, University of Sassari, 07100 Sassari, Italy; mscaglione@uniss.it

**Keywords:** breast density, background parenchymal enhancement, contrast-enhanced mammography, BI-RADS, risk stratification, standardized lexicon, BPE-CEM Standard Scale (BCSS), Relational Database Management System (RDBMS), applied regression analysis

## Abstract

**Introduction**: Breast density is a well-recognized factor in breast cancer risk assessment, with higher density linked to increased malignancy risk and reduced sensitivity of conventional mammography. Background parenchymal enhancement (BPE), observed in contrast-enhanced imaging, reflects physiological contrast uptake in non-pathologic breast tissue. While extensively characterized in breast MRI, the role of BPE in contrast-enhanced mammography (CEM) remains uncertain due to inconsistent findings regarding its correlation with breast density and cancer risk. Unlike breast density—standardized through the ACR BI-RADS lexicon—BPE lacks a uniform classification system in CEM, leading to variability in clinical interpretation and research outcomes. To address this gap, we introduce the BPE-CEM Standard Scale (BCSS), a structured four-tiered classification system specifically tailored to the two-dimensional characteristics of CEM, aiming to improve consistency and diagnostic alignment in BPE evaluation. **Materials and Methods:** In this retrospective single-center study, 213 patients who underwent mammography (MG), ultrasound (US), and contrast-enhanced mammography (CEM) between May 2022 and June 2023 at the “A. Perrino” Hospital in Brindisi were included. Breast density was classified according to ACR BI-RADS (categories A–D). BPE was categorized into four levels: Minimal (< 10% enhancement), Light (10–25%), Moderate (25–50%), and Marked (> 50%). Three radiologists independently assessed BPE in a subset of 50 randomly selected cases to evaluate inter-observer agreement using Cohen’s kappa. Correlations between BPE, breast density, and age were examined through regression analysis. **Results:** BPE was Minimal in 57% of patients, Light in 31%, Moderate in 10%, and Marked in 2%. A significant positive association was found between higher breast density (BI-RADS C–D) and increased BPE (*p* < 0.05), whereas lower-density breasts (A–B) were predominantly associated with minimal or light BPE. Regression analysis confirmed a modest but statistically significant association between breast density and BPE (R^2^ = 0.144), while age showed no significant effect. Inter-observer agreement for BPE categorization using the BCSS was excellent (κ = 0.85; 95% CI: 0.78–0.92), supporting its reproducibility. **Conclusions:** Our findings indicate that breast density is a key determinant of BPE in CEM. The proposed BCSS offers a reproducible, four-level framework for standardized BPE assessment tailored to the imaging characteristics of CEM. By reducing variability in interpretation, the BCSS has the potential to improve diagnostic consistency and facilitate integration of BPE into personalized breast cancer risk models. Further prospective multicenter studies are needed to validate this classification and assess its clinical impact.

## Simple Summary

Contrast-enhanced mammography (CEM) is increasingly used in breast cancer diagnostics, especially for women with dense breast tissue. However, interpreting background parenchymal enhancement (BPE)—a normal tissue reaction to contrast—is still subjective due to the lack of a standardized system. This study introduces the Breast Contrast Standard Scale (BCSS), a new four-level system designed specifically for CEM to assess BPE consistently. The results show that higher breast density is linked to stronger BPE, and the proposed scale demonstrated excellent agreement between different radiologists. Standardizing BPE assessment could improve diagnostic accuracy and support personalized screening strategies in clinical practice.

## 1. Introduction

Breast density is a well-recognized risk factor for breast cancer, correlating not only with increased malignancy risk but also with reduced sensitivity of conventional mammography [1,2]. Background parenchymal enhancement (BPE)—defined as the physiological uptake of contrast in non-pathological fibroglandular tissue—has emerged as a potential imaging biomarker in contrast-enhanced breast imaging. While extensively studied in breast MRI, the clinical significance of BPE remains controversial, with evidence pointing in divergent directions—some identifying it as a risk biomarker, others finding limited predictive value [3,4]. This underscores the importance of modality-specific classification systems that account for both technical constraints and interpretive requirements unique to CEM.

Contrast-enhanced mammography (CEM) is an emerging hybrid modality that combines anatomical and functional imaging, offering advantages over MRI such as greater accessibility, lower cost, and faster acquisition [5,6]. BPE can be visualized on CEM, but its assessment is complicated by the planar, two-dimensional nature of the modality, which lacks the volumetric and kinetic resolution available with MRI. Notably, pronounced BPE in CEM can mask lesions and reduce diagnostic accuracy [7,8]. Unlike breast density, which is systematically categorized via the ACR BI-RADS lexicon [9], there is currently no standardized lexicon or scoring system for BPE in CEM, resulting in interpretive variability and limited comparability across studies.

The relationship between BPE and breast density remains controversial. Some evidence suggests a positive correlation [10,11], while other studies report no significant link [12,13]. These inconsistencies highlight the need for a dedicated classification system tailored specifically to the technical and interpretive characteristics of CEM. Importantly, existing BPE grading schemes developed for MRI—such as that by Sorin et al. [9]—are based on volumetric and temporal contrast dynamics not available in CEM, making direct transposition impractical.

Unlike MRI-based frameworks, the Breast Contrast Standard Scale (BCSS) was developed specifically for CEM. It defines enhancement based on semi-quantitative thresholds of parenchymal involvement (e.g., <10% for Minimal), complemented by anatomical criteria such as masking of ducts and vessels. This modality-specific approach reflects the practical constraints of CEM interpretation and offers a novel step toward standardizing BPE assessment in clinical and research settings.

Recent advances in this field have also led to ongoing investigations exploring artificial intelligence—particularly neural networks—to enhance standardization and reduce variability in the assessment of BPE and breast density. Preliminary results from a complementary study using AI have shown promising improvements in assisting radiologists with borderline BI-RADS C and D cases, where inter-reader variability is greatest. Methodological details and early findings from this AI-based approach are reported separately and will support the clinical integration of such tools.

### 1.1. Study Objective

This study evaluates the relationship between breast density and background parenchymal enhancement (BPE) in contrast-enhanced mammography (CEM) and introduces the Breast Contrast Standard Scale (BCSS)—a four-level classification system (Minimal, Light, Moderate, Marked), designed to standardize BPE reporting in CEM.

The BCSS adapts MRI-derived grading concepts to the constraints of CEM by integrating enhancement thresholds and anatomical distribution criteria suitable for planar imaging. Specifically, the scale combines semi-quantitative estimates of parenchymal enhancement (e.g., < 10% for Minimal) with anatomical markers such as the masking of ducts or vascular structures.

### 1.2. The BCSS Aims to:

- Improve inter-reader agreement in BPE interpretation on CEM.
- Enable reproducible comparisons across imaging studies and centers.
- Facilitate the inclusion of BPE in structured breast cancer risk stratification frameworks.

This classification system represents a clinically relevant step toward reducing subjectivity in CEM interpretation and may support both diagnostic accuracy and personalized screening strategies for women with dense breasts.

## 2. Materials and Methods

This retrospective single-center study was conducted at the Interventional Senology Unit of P.O. “A. Perrino” Hospital, Brindisi, Italy, from May 2022 to June 2023, following Good Clinical Practice guidelines. Among 314 initially evaluated patients, 213 women aged 28–80 years met inclusion criteria [Table 1]. Eligible subjects presented with suspicious breast lesions classified as BI-RADS 4 or 5 on CEM and underwent complete diagnostic workup including ultrasound (US), conventional mammography (MG), and contrast-enhanced mammography (CEM) prior to biopsy and histological confirmation of invasive breast cancer [Table 2]. Due to its retrospective nature, no additional consent beyond routine imaging authorization was required.

### 2.1. Data Management

Clinical and imaging data were entered into three structured relational databases: demographics (patient ID, birth date), imaging parameters (breast density per ACR BI-RADS A-D, BPE categories Minimal/Light/Moderate/Marked, completion status of US, MG, and CEM), and morphometric glandular measurements in millimeters across four fields.

The design and implementation of these relational databases, as well as the methods used for data analysis, followed established standards in database management and statistical methodology [14,15,16,17,18,19,20,21]. 

### 2.2. Inclusion and Exclusion Criteria

Inclusion: Women ≥ 18 years with suspicious findings (BI-RADS 4 or 5) on CEM.

Exclusion: Contrast contraindications (pregnancy, allergy, renal insufficiency per ESUR guidelines), prior breast cancer, breast implants, ongoing neoadjuvant therapy, incomplete imaging, absent histologic confirmation, or biopsy/radiotherapy within 21 days before CEM.

### 2.3. CEM Protocol

Contrast Administration

After screening for contraindications, Iohexol 350 mg I/mL (Omnipaque^®^, GE Healthcare, Chicago, IL, USA) was injected intravenously at 1.5 mL/kg via 20-gauge catheter at 3 mL/s, followed by 20 mL saline flush.

### 2.4. Image Acquisition

CEM was performed using a full-field digital mammography system (Senographe Pristina with SenoBright^®^ RS26.2 software, GE Healthcare, Chicago, IL, USA). Bilateral craniocaudal (CC) and mediolateral oblique (MLO) views were acquired under compression. Each breast was imaged using low-energy (26–31 keV) and high-energy (45–49 keV) exposures, beginning 2 min post injection, with acquisition lasting ~ 1.5 s per view. Total exam time was under 7 min. Images were recombined to produce subtraction images for BPE evaluation.

### 2.5. Image Interpretation and BPE Assessment

Breast density was assessed on low-energy images using ACR BI-RADS v5 criteria [9]. BPE was evaluated on recombined subtraction images using the newly proposed BPE-CEM Standard Scale (BCSS), which defines four BPE levels based on semi-quantitative and qualitative criteria:

- Minimal (MIN): <10% of visible fibroglandular tissue enhanced, faint enhancement not obscuring ducts/vessels.
- Light (LIE): 10–25% enhancement, mild masking but key anatomical landmarks visible.
- Moderate (MOD): 25–50% enhancement, partial overlap/obscuration of ducts, vessels, and glandular architecture, potentially interfering with lesion visibility.
- Marked (MAR): >50% enhancement, strong masking/obscuration complicating lesion detection.

Experienced breast radiologists visually estimated enhancement extent via standardized scoring sheets, cross-referencing low-energy images to define glandular boundaries. This combined quantitative and anatomical approach aims to enhance reproducibility tailored to CEM’s 2D nature.

### 2.6. Inter-Observer Agreement Assessment

Three expert breast radiologists (>10 years of experience) independently scored a randomized subset of 50 cases. Agreement was quantified using Cohen’s kappa statistic, interpreted according to the Landis and Koch criteria.

### 2.7. Statistical Analysis

Data were managed using a relational database management system (DBMS) to minimize redundancy and ensure consistency. Descriptive statistics summarized patient age, BPE, and breast density distributions. Lesion size comparisons were performed using the Bland–Altman method. A multiple linear regression analysis was performed with BPE as the dependent variable and breast density and age as independent predictors, in order to assess their explanatory power.

Inter-observer agreement for BPE classification was assessed by three radiologists independently reviewing a random sample of 50 cases, with agreement quantified using Cohen’s kappa statistic.

## 3. Results

### 3.1. Patient Age Distribution

The study population ranged from 28 to 79 years, with a peak incidence around age 60—consistent with typical breast imaging cohorts.

BPE Categorization

Among the 213 patients included:

- Minimal BPE: 57%.
- Light BPE: 31%.
- Moderate BPE: 10%.
- Marked BPE: 2%.

This distribution reflects a predominance of low-grade enhancement in the study population.

### 3.2. Inter-Observer Agreement

BPE classification using the BCSS showed near-perfect inter-observer agreement, with a Cohen’s kappa of 0.85 (95% CI: 0.78–0.92). This supports the reproducibility and operational feasibility of the proposed scale.

### 3.3. Breast Density and Imaging Modality

A total of 313 imaging studies (pre- and post-contrast) were analyzed:

- Non-contrast (based on low-energy images): 11% A, 29% B, 26% C, 17% D.
- Contrast-enhanced (CEM): 1% A, 7% B, 4% C, 4% D.

This contrast-induced shift in perceived density corresponds to enhanced conspicuity of fibroglandular tissue due to its physiological vascularization and dynamic contrast uptake. This phenomenon represents a genuine physiological imaging effect inherent to contrast-enhanced mammography, distinct from imaging artifacts, and should be considered when interpreting breast density changes post-contrast administration.

### 3.4. Age-Based Stratification of BPE

BPE showed an inverse relationship with age:

- Age 25–40: 5% Minimal/Light, 1% Moderate/Marked.
- Age 41–55: 38% Minimal/Light, 6% Moderate/Marked.
- Age > 55: 46% Minimal/Light, 4% Moderate/Marked.

These findings align with physiological estrogen decline and support previously described age-dependent trends in background enhancement.

### 3.5. S Metric Analysis

The S-value is a statistical metric quantifying the difference between pre- and post-contrast signal intensity distributions within fibroglandular tissue, providing an objective estimate of the extent and variability of BPE and complementing the semi-quantitative evaluation by enhancing precision and reproducibility in assessing parenchymal enhancement.

- Density A: 14% of cases; 3 with S < −2, 2 with S > 2.
- Density B: 36%; 7 with S < −2, 8 with S > 2.
- Density C: 31%; 7 each with S < −2 and S > 2.
- Density D: 19%; 7 with S < −2, 3 with S > 2.

These findings suggest intra-group heterogeneity, indicating that glandular morphology may add nuance to standard density classifications.

### 3.6. Regression Analysis

A multiple linear regression analysis was conducted to explore the relationship between breast density, BPE, and age [Table 3]:

- Multiple R: 0.38 (moderate correlation).
- R^2^: 0.144 (14.4% of variance explained).
- Adjusted R^2^: 0.136.
- Standard error: 0.8639.
- BPE showed a statistically significant positive association with breast density (*p* < 0.05).
- Age was not a significant predictor (*p* = 0.14).

**Table 3:** This table presents the results of the ANOVA (Analysis of Variance) for the regression model. The table shows the degrees of freedom (df), sum of squares (SS), mean square (MS), F-statistic, and significance value for the regression and residual components, along with the total sum of squares. The very low significance F (7.8594 × 10^−8^) indicates that the model is statistically significant.

ANOVA (Analysis of Variance)					
Source	df	SS	MS	F	Significance F
Regression	2	26.42365998	13.21182999	17.70217459	7.8594 × 10^−8^
Residual	210	156.7312696	0.746339379		
Total	212	183.1549296			

Although BPE shows a statistically significant correlation with breast density (*p* < 0.05), the relatively low R^2^ value (0.144) indicates modest predictive power, suggesting that additional non-linear or latent variables likely influence BPE [Table 4].

**Figure 1:**
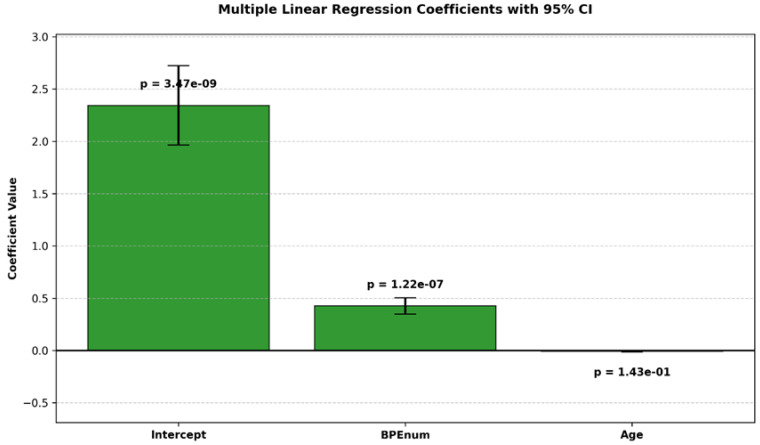
Bar plot of estimated coefficients from a multiple linear regression model with 95% confidence intervals. *p*-values are shown above each bar.

The estimated coefficients and their confidence intervals are illustrated in Figure 1.

## 4. Discussion

The interplay between background parenchymal enhancement (BPE) and breast density has been extensively investigated in the literature, yet remains controversial, with conflicting evidence regarding their correlation and individual roles in breast cancer risk stratification [22,23]. BPE, initially described in contrast-enhanced breast MRI, reflects physiologic contrast uptake in non-pathologic fibroglandular tissue and is influenced by hormonal status, vascular perfusion, and parenchymal composition [24]. While several studies have proposed BPE as an independent imaging biomarker for breast cancer risk, findings have been inconsistent or inconclusive [25].

Breast density, by contrast, is a well-established and independent risk factor for breast cancer. Dense breast tissue not only correlates with increased malignancy rates but also reduces the sensitivity of standard mammography [26,27]. However, the relationship between BPE and density—particularly in the setting of contrast-enhanced mammography (CEM)—remains poorly defined. One major challenge is the absence of a standardized BPE classification system tailored specifically for CEM, unlike MRI which benefits from a well-established volumetric lexicon [28,29,30].

CEM is increasingly recognized as a clinically valuable alternative to breast MRI, combining anatomical and functional information with greater accessibility and shorter acquisition times. However, BPE as visualized in CEM differs from MRI due to the modality’s planar, two-dimensional nature and its reliance on recombined subtraction imaging. These technical differences complicate BPE interpretation and reduce reproducibility across clinical settings. Additionally, whereas breast density is categorized systematically using the ACR BI-RADS lexicon, no universally accepted classification currently exists for BPE in CEM, contributing to interpretive variability and limiting research comparability.

Our study addresses this gap by introducing the BPE-CEM Standard Scale (BCSS), a novel four-level classification (Minimal, Light, Moderate, Marked) based on semi-quantitative enhancement thresholds and anatomical criteria. This modality-specific scale is designed to standardize BPE assessment within the constraints of CEM’s 2D imaging format.

Preliminary data from a subsequent analysis on the same dataset suggest that artificial intelligence (AI), particularly neural networks, can reduce inter-observer variability in cases with borderline BI-RADS classifications (C and D), where subjectivity is most pronounced. The lower MAE observed with neural networks indicates improved predictive precision over traditional linear regression models, aligning with the established utility of backpropagation and gradient-based optimization in modeling complex biomedical data. These findings resonate with the foundational work of Rosenblatt (1958), Rumelhart et al. (1986), and Goodfellow et al. (2016), which collectively underscore the power of iterative weight optimization via backpropagation in enhancing predictive accuracy [31,32,33].

Taken together, these findings reinforce the importance of a standardized BPE lexicon tailored to CEM and open promising avenues for the integration of AI in breast imaging protocols. Further validation is needed, but this dual approach—structural standardization through BCSS and variability reduction via AI—may significantly improve diagnostic reproducibility in women with dense breasts.

### 4.1. Key Findings

- BPE distribution: Minimal in 57% of patients, Light in 31%, Moderate in 10%, and Marked in 2%.
- Density correlation: Higher breast density categories (BI-RADS C–D) were significantly associated with Moderate-to-Marked BPE, whereas lower densities (A–B) correlated with Minimal-to-Light BPE (*p* < 0.05).
- Regression analysis: Demonstrated a statistically significant association between BPE and breast density (R^2^ = 0.144), with a moderate multiple correlation coefficient (R = 0.38). Age was not a significant predictor (*p* = 0.14).
- Inter-observer agreement: The BCSS showed excellent reproducibility, with Cohen’s κ = 0.85 (95% CI: 0.78–0.92), supporting its feasibility and consistency in clinical practice.

Although the regression model confirmed a statistically significant association between BPE and breast density, the low R^2^ value (0.144) reflects limited explanatory power, suggesting that breast density alone accounts for only a small portion of the variance in BPE. Unmeasured biological and clinical factors—such as hormonal status, menstrual phase, BMI, or genetic predisposition—may act as latent confounders. Incorporating these variables, along with non-linear modeling approaches, may improve future predictive accuracy [3,4].

Moreover, the standard error (0.8639) and Mean Absolute Error (MAE) reflect residual variance, underscoring the limited precision of the current model. While the overall regression was statistically significant (F = 17.70, *p* < 0.001), the individual contribution of breast density remains modest.

### 4.2. Introducing the BCSS: Toward Standardization

The proposed BCSS offers a practical solution for harmonizing BPE assessment in CEM. Unlike MRI-based systems that rely on volumetric and temporal enhancement criteria, the BCSS adapts semi-quantitative thresholds to the two-dimensional nature of CEM and incorporates anatomical indicators such as ductal and vascular masking.

For instance, “Moderate” BPE in CEM refers to enhancement of 25–50% of fibroglandular tissue with partial obscuration of key structures, which—despite lacking volumetric context—may still compromise lesion visibility in planar imaging. This distinction is critical for aligning classification with diagnostic performance in CEM.

The high inter-observer agreement observed in our study confirms the reliability of the BCSS, suggesting that it may enhance both diagnostic consistency and research comparability across institutions. However, its clinical utility remains to be confirmed through multicenter, prospective validation studies encompassing diverse patient populations and imaging platforms.

### 4.3. Clinical Integration and Operational Implementation of the BCSS

The clinical implementation of the BCSS requires a series of operational steps aimed at ensuring its applicability, standardization, and reliability in routine practice. The integration of the scale into DCE-MRI reporting protocols is essential, supported by targeted radiologist training focused on the semi-quantitative assessment of BPE extent and anatomical masking criteria. The development of digital tools that can be integrated within PACS systems is necessary to standardize data collection. Multicenter validation is crucial to ensure robustness across different platforms and clinical populations, facilitating routine adoption. Future integration with artificial intelligence algorithms could reduce inter-operator variability and enhance decision support, consolidating the BCSS as a reliable parameter for risk stratification and personalized management of patients with dense breasts.

### 4.4. Limitations and Future Directions

This study has several limitations. Its retrospective, single-center design may limit generalizability. Additionally, the lack of clinical data on hormonal status, BMI, menstrual cycle phase, and endocrine therapies restricted our ability to account for confounding variables that may influence BPE. Moreover, the study population was drawn from a diagnostic rather than a screening cohort, potentially affecting the applicability of findings to broader populations. All imaging was also acquired using a single CEM device (GE Senographe Pristina, GE Healthcare, Chicago, IL, USA) and a fixed contrast protocol, which may limit reproducibility across different vendors or acquisition settings.

To strengthen the clinical impact of the BCSS and deepen understanding of BPE’s biological and imaging correlates, future research should focus on the following:

- Prospective, multicenter validation of the BCSS across different imaging platforms.
- Integration of AI-based tools for objective, automated quantification of BPE, reducing reader subjectivity.
- Incorporation of hormonal, genetic, and physiological variables into risk prediction models.
- Application of machine learning and deep learning methods to uncover complex, non-linear associations and enhance predictive accuracy.

In particular, neural networks may minimize prediction error (e.g., MAE), uncover latent patterns, and improve the integration of BPE into individualized risk models and screening pathways—especially in women with dense breasts, where conventional mammography is limited. This study represents an initial step toward future quantitative standardization of BPE assessment; however, it does not yet incorporate advanced predictive modeling, which will be addressed in separate analyses.

Finally, while the exclusion of patients with prior breast cancer, breast implants, neoadjuvant therapy, or incomplete imaging was intended to reduce confounding factors—such as post-treatment fibrosis, vascular remodeling, and artifact-induced distortion—this methodological rigor may also limit the generalizability of our findings. Future studies should address these specific populations to assess the broader applicability of the BCSS.

Complementary exploratory analyses—conducted on a subset of the same dataset but not included in the present study—demonstrated that deep neural network models significantly reduced prediction error compared to linear regression (MAE = 0.691 vs. 0.8639). This finding suggests a superior ability of AI-based approaches to capture complex, non-linear relationships between breast density and BPE. The systematic application and validation of these methods will be addressed in Part 2 of this research, representing a promising direction for enhancing predictive performance and enabling more personalized risk stratification

## 5. Conclusions

In the setting of contrast-enhanced mammography (CEM), background parenchymal enhancement (BPE) remains a poorly standardized yet clinically relevant imaging feature. Our proposed BPE-CEM Standard Scale (BCSS) offers a structured and modality-specific framework to improve the consistency of BPE evaluation, particularly in women with dense breasts, where diagnostic interpretation is most challenging.

While our findings confirm a modest correlation between breast density and BPE, the limited explanatory power of linear models highlights the complexity of this relationship. These results support the need for further methodological innovation to capture the nuanced interplay between tissue composition and enhancement patterns.

Preliminary explorations with artificial intelligence (AI), including neural networks, suggest potential in reducing inter-reader variability and aiding radiologists in ambiguous cases. Although these computational tools require further validation, they may serve as valuable adjuncts in enhancing diagnostic consistency and enabling personalized risk stratification.

Future efforts should aim to integrate probabilistic AI models and multimodal data—including hormonal, genetic, and physiological variables—into predictive frameworks. This would enhance both the interpretability and clinical relevance of BPE as an imaging biomarker. Importantly, these technological advancements must remain adjunctive, reinforcing rather than replacing the nuanced judgment of experienced radiologists.

## Figures and Tables

**Table 1:** Age group, record number, percentage, and density distribution by BPE.

Age Group	Number of Record (out of 268)	Percentage	BPE	Notes on Density
25–40	11	5%	MIN/LIE	
25–40	2	1%	MOD/MAR	No A and no D
41–55	80	38%	MIN/LIE	
41–55	13	6%	MOD/MAR	No A
Over 55	97	46%	MIN/LIE	
Over 55	9	4%	MOD/MAR	No A, one B and one D

**Table 2:** Count of records by letter, with percentages and details on the S value.

Letter	Count	Percentage (%)	Null Values	Details on S Value
A	18	14%	13	3 records with S < −2, 2 with S > 2
B	47	36%	31	7 records with S < −2, 8 with S > 2
C	40	31%	25	7 records with S < −2, 7 with S > 2
D	25	19%	14	7 records with S < −2, 3 with S > 2

**Table 4:** The table reports the estimated coefficients, standard errors, *t*-statistics, *p*-values, and 95% confidence intervals for the model parameters.

	Coefficients	Standard Error	*t* Stat	*p*-Value	Lower 95%	Upper 95%	Lower 95.0%	Upper 95.0%
Intercept	2.343692153	0.379884019	6.169493937	3.47353 × 10^−9^	1.594817367	3.092566939	1.594817367	3.092566939
BPEnum	0.426517913	0.077858245	5.478134158	1.22301 × 10^−7^	0.273034024	0.580001803	0.273034024	0.580001803
Age	0.008787179	0.005970236	1.471831024	0.14256352	0.020556454	0.002982096	0.020556454	0.002982096

## Data Availability

The data supporting the findings of this study are available within the article.

## Abbreviations

The following abbreviations are used in this manuscript:

BPE	Background Parenchymal Enhancement
CEM	Contrast-Enhanced Mammography
BCSS	BPE-CEM Standard Scale
BI-RADS	Breast Imaging Reporting and Data System
MAE	Mean Absolute Error
DBMS	Database Management System
AI	Artificial Intelligence
